# Effect of a Mobile App–Based Urinary Incontinence Self-Management Intervention Among Pregnant Women in China: Pragmatic Randomized Controlled Trial

**DOI:** 10.2196/43528

**Published:** 2023-06-27

**Authors:** Ling Chen, Danli Zhang, Tiantian Li, Sha Liu, Jie Hua, Wenzhi Cai

**Affiliations:** 1 Department of Nursing Shenzhen Hospital, Southern Medical University Shenzhen China; 2 School of Nursing, Southern Medical University Guangzhou China

**Keywords:** mobile health, mHealth, mobile apps, urinary incontinence, pregnancy, pragmatic randomized controlled trial, mobile phone

## Abstract

**Background:**

Urinary incontinence (UI) is a highly prevalent health concern commonly observed during and after pregnancy that can substantially impact women’s physical and psychological well-being and quality of life. Owing to its numerous advantages, mobile health may be a promising solution; however, it is unclear whether the app-based intervention can effectively improve UI symptoms during and after pregnancy.

**Objective:**

This study aimed to evaluate the effectiveness of the Urinary Incontinence for Women (UIW) app–based intervention for UI symptom improvement among pregnant women in China.

**Methods:**

Singleton pregnant women without incontinence before pregnancy who were aged ≥18 years and between 24 and 28 weeks of gestation were recruited from a tertiary public hospital in China and were randomly allocated (1:1) to either an experimental group (n=63) or a control group (n=63). The experimental group received the UIW app intervention and oral pelvic floor muscle training (PFMT) instructions, whereas the control group received oral PFMT instructions alone. Neither the participants nor the researchers were blinded to the intervention. The primary outcome was UI severity. The secondary outcomes included quality of life, self-efficacy with PFMT, and knowledge of UI. All data were collected at baseline, 2 months after randomization, and 6 weeks post partum through electronic questionnaires or by checking the electronic medical record system. Data analysis followed the intention-to-treat principle. A linear mixed model was used to examine the intervention effect on primary and secondary outcomes.

**Results:**

Participants in the experimental and control groups were comparable at baseline. Of the 126 overall participants, 117 (92.9%) and 103 (81.7%) women completed follow-up visits at 2 months after randomization and 6 weeks after delivery, respectively. A statistically significant difference in UI symptom severity was observed between the experimental group and control group (2 months after randomization: mean difference −2.86, 95% CI −4.09 to −1.64, *P*<.001; 6 weeks post partum: mean difference −2.68, 95% CI −3.87 to −1.49, *P*<.001). For the secondary outcomes, a statistically significant intervention effect on the quality of life, self-efficacy, and UI knowledge was found at the 2-month follow-up (all *P*<.05) and 6 weeks post partum (all *P*<.001).

**Conclusions:**

The app-based UI self-management intervention (UIW) effectively improved UI symptom severity, quality of life, self-efficacy with PFMT, and knowledge of UI during the late pregnancy and early postnatal periods. Larger multicenter studies with a longer postpartum follow-up are required to further extend these findings.

**Trial Registration:**

Chinese Clinical Trial Registry ChiCTR1800016171; http://www.chictr.org.cn/showproj.aspx?proj=27455

**International Registered Report Identifier (IRRID):**

RR2-10.2196/22771

## Introduction

Urinary incontinence (UI), defined as the involuntary leakage of urine [1], is a prevalent health concern among women that negatively affects their quality of life and mental well-being [2-4]. Pregnancy and childbirth are well-known risk factors for UI [5-7]. Approximately 41% of women present with UI during pregnancy [8], and the prevalence of UI in the first year post partum has been reported to increase from 24% at 6 weeks to 32% at 12 months post partum [9]. Even worse, approximately two-fifths of women continue to experience UI for 12 years after birth [10], although UI is a dynamic condition that may spontaneously remit over time [11-13]. Therefore, early prevention and treatment of UI during pregnancy are of utmost importance.

Pelvic floor muscle training (PFMT) is the first-line conservative treatment for UI [14]. The latest Cochrane systematic review recommends antenatal PFMT for all childbearing women regardless of continence status [15]. However, although the effectiveness of PFMT is well-documented, few women engage in PFMT during pregnancy [16-18]. The barriers to deflecting pregnant women from performing PFMT are multifaceted, such as misconceptions about UI as a normal physiological phenomenon in pregnancy, limited knowledge about PFMT, forgetfulness, and lack of time [19,20]. In addition, because of the increasing workload and time constraints, obstetric health care professionals have limited opportunities to deliver perinatal incontinence care [21,22]. Even worse, PFMT instructions are not yet considered a common practice for routine antenatal care services in China [23]. Furthermore, during the COVID-19 pandemic, conservative face-to-face treatment for UI has been difficult to organize. Considering these challenges, effective strategies for improving health care delivery and services for UI are urgently needed.

In recent years, an increasing body of research has demonstrated that mobile health (mHealth) interventions may benefit health promotion, especially in chronic illness management areas [24,25]. With the widespread use of smartphones [26], mobile apps have emerged as a promising strategy to assist in the self-management of UI. Relevant research has reported that women with UI have a positive attitude toward mHealth, as this new technology increases their access to care, preserves their privacy, enables them to schedule the exercises more flexibly, and offers reminders to continue with their PFMT, thus lowering barriers to treatment [27-29]. More importantly, several studies have found that mobile apps could greatly improve UI outcomes [30-32]. However, prior studies on the utility of app-based interventions for improving UI symptoms have predominantly focused on middle-aged and older women or primiparas women [32,33], and available evidence on the effect in antenatal women is scarce.

Herein, we developed a Urinary Incontinence for Women (UIW) mobile app for maternal UI self-management [34]. This study aimed to examine the effectiveness of UIW app–based intervention in improving UI symptoms among pregnant women in China. We hypothesized that the experimental group receiving the UIW app intervention and oral PFMT instructions would experience greater improvement in UI symptoms at follow-up compared with the control group receiving only oral PFMT instructions.

## Methods

### Study Design

This was a single-center, 2-arm, unblinded pragmatic randomized controlled trial with 1:1 intervention allocation that was reported in line with the CONSORT-eHEALTH (Consolidated Standards of Reporting Trials of Electronic and Mobile Health Applications and Online Telehealth) guidelines (Multimedia Appendix 1) [35].

### Ethics Approval

The study was registered in the Chinese Clinical Trial Registry (ChiCTR1800016171), and the ethical approval was obtained from the Ethics Committees of Shenzhen Hospital, Southern Medical University (NYSZYYEC20190012). The trial protocol has been published, and the findings from an embedded process evaluation will be presented elsewhere [34].

### Participants

A trained graduate nurse sequentially recruited participants during routine obstetrics clinic visits at Shenzhen Hospital, Southern Medical University, a tertiary level A public hospital in China, between June and October 2020. Eligibility was first assessed by checking the medical records from the obstetrics clinic, after which those who met the eligibility criteria (see the following paragraph) were provided with an information letter describing the study and invited to participate in the study. After providing written informed consent, eligible participants completed a baseline electronic survey at the same time. They were told to contact the researchers via telephone if they experienced any discomfort or pain associated with the PFMT. After completing all follow-ups, participants received compensation with a gift worth RMB ¥100 (equal to US $14.44).

The inclusion criteria were as follows: (1) age ≥18 years, (2) having a singleton pregnancy, (3) being at 24 to 28 gestational weeks, (4) being continent before pregnancy, and (5) having a mobile phone with internet access. The exclusion criteria were (1) cognitive impairment and psychiatric conditions, (2) a history of pelvic organ prolapse or pelvic surgery, (3) severe comorbidities found in prenatal examinations that were not suitable for PFMT in pregnancy (such as heart disease, pregnancy-induced hypertension, diabetes mellitus, threatened abortion, placenta previa, placental abruption, and fetal growth restriction), and (4) pain during pelvic floor muscle contraction.

### Randomization and Masking

The trial used a simple randomization approach. A 1:1 random assignment sequence was generated via a table of random numbers by a research assistant who was not involved in the study and was placed into sequentially numbered, opaque, and sealed envelopes. When each participant was enrolled, the intervention manager opened the envelope in sequence, and the participant was assigned to the experimental group, which received oral PFMT instructions and the UIW app intervention, or the control group, which received oral PFMT instructions. Owing to the nature of the app-based intervention and self-reported outcomes, neither the participants nor the research staff were blinded to the intervention.

### Interventions

#### UIW App

The participants allocated to the experimental group were granted access to the UIW app, which is a mobile app for Chinese pregnant women developed by our research team with technical assistance from the Guangdong Zhuoshang Network Technology Company (registration number: 2019SR1342273). The introduction of the UIW app, including its content and function, can be found in previously published papers [34]. Briefly, the app focuses on the PFMT module, where a staged training program is designed in the order of difficulty. It consists of 2 test versions and 4 training versions, and the specific program components are presented in Textbox 1. Participants could choose an affordable training version according to the mastery level achieved during the evaluation of the test version. In addition, when performing PFMT, the app would show real-time, dynamic guidance in columnar graphics, presenting the duration and intensity of pelvic floor muscle contraction with concomitant relaxation. The app also contains other modules, including Risk Assessment, Health Education, and an Online Evaluation forum, with the functions of education, reminders, consultation, self-monitoring, and others. First, the pregnant women were guided by 2 researchers to download the free UIW app on their phones from the Apple App Store or by scanning a QR code. After registering and activating accounts, the researchers explained the functions of the app in detail to participants and demonstrated how to use it. For example, participants were individually instructed on how to follow the animations and sound commands to contract and relax the pelvic floor muscles in the PFMT forum or browse the articles in the Health Education forum. Once the study started, the participants could log in and use the app autonomously. In addition, the app would automatically provide reminders 3 times daily to prompt participants to engage in exercise.

**Contents of staged pelvic floor muscle training program in the Urinary Incontinence for Women app (test contraction: 2-second contraction and 2-second relaxation; strong contraction: 6-second contraction and 6-second relaxation; and rapid contraction: 3-second contraction and 3-second relaxation).**

**Test version**

- Stage 1: eight test contractions for 1 set of exercises, 3 sets per day
- Stage 2: eight test contractions+2 strong contractions for 1 set of exercises, 3 sets per day

**Training version**

- Stage 1: eight strong contractions+1 endurance contraction for 1 set of exercises, 3 sets per day; endurance contraction at this stage was 15-second contraction and 5-second relaxation
- Stage 2: ten strong contractions+1 endurance contraction for 1 set of exercises, 3 sets per day; endurance contraction at this stage was 25-second contraction and 5-second relaxation (if pregnant women cannot reach this training stage as assessed, stay in the previous stage)
- Stage 3: ten strong contractions+1 endurance contraction+5 rapid contractions for 1 set of exercises, 3 sets per day, endurance contraction at this stage was 35-second contraction and 35-second relaxation (if pregnant women cannot reach this training stage as assessed, stay in the previous stage)
- Stage 4: ten strong contractions+1 endurance contraction+10 strong contraction+5 rapid contractions for 1 set of exercises, 3 sets per day; endurance contraction at this stage was 35-second contraction and 35-second relaxation (if pregnant women cannot reach this training stage as assessed, stay in the previous stage)

#### Oral PFMT Instructions

Both groups received oral PFMT instructions, comprising one-to-one health education about UI and PFMT practice guidance at the time of recruitment by experienced obstetricians, without further follow-up treatments. The health education content covered the following 5 topics: introduction to UI and lifestyle factors associated with UI (weight management, constipation prevention, pelvic floor muscle care, and general antenatal care), which was consistent with the information in the Health Education forum of the UIW app. When teaching PFMT-related skills, pregnant women were directed to lie in the supine position, with the abdomen and buttocks muscles relaxed and selectively contracted and relaxed the muscles around the urethra, vagina, and anus. During this time, PFMT guidance was provided similar to that in stage 1 of the training version in the PFMT program of the UIW app. Obstetricians placed one hand on the abdomen and the other on the perineal body to confirm whether the women had correctly mastered the exercise technique. The general goal of the training was to exercise 3 times a day for at least 2 months.

### Outcomes and Measures

#### Baseline Measures

At baseline, participant demographic characteristics and pregnancy-related data, such as age, education, height, prepregnancy weight, number of pregnancies, and prior abortions, were collected through a self-designed electronic questionnaire (by scanning a QR code on the smartphone) before randomization. In addition, delivery information, including gestational age of delivery, birth mode, perineal injury, and neonatal weight, was obtained by checking the electronic medical record system after delivery.

All outcomes were measured at baseline, 2 months after randomization, and 6 weeks post partum. The control group completed follow-up assessments electronically (via a QR code linked to questionnaires), whereas the experimental group completed the questionnaires provided in the Online Evaluation forum of the UIW app.

#### Primary Outcome

The primary outcome was the severity of incontinence symptoms based on the International Consultation on Incontinence Questionnaire-Urinary Incontinence Short Form (ICIQ-UI-SF) [36], assessed at baseline, 2 months after randomization, and 6 weeks post partum. The Chinese version of the ICIQ-UI-SF is a 4-item instrument with 3 items used to assess the frequency of leakage, amount of leakage, and its impact on quality of life and 1 unscored item diagnosing the type of UI, which has shown adequate internal consistency and reliability [37]. The total score on the ICIQ-UI-SF ranges from 0 to 21, with 0 to 7 indicating mild symptoms, 8 to 13 indicating moderate symptoms, and 14 to 21 indicating severe symptoms.

#### Secondary Outcomes

The secondary outcomes were quality of life, self-efficacy with PFMT, and knowledge of UI, measured at baseline, 2 months after randomization, and 6 weeks post partum.

Quality of life was measured using the Incontinence Impact Questionnaire-7 (IIQ-7), with 7 items assessing 4 domains (physical activity, travel, social activities, and emotional health) [38]. The IIQ-7 score ranges from 0 to 21, with higher scores indicating a greater impact on life. The Chinese version of the IIQ-7 has a Cronbach α coefficient of .824 and a high construct validity [39].

The self-efficacy of PFMT was assessed using the 23-item Broome Pelvic Muscle Self-Efficacy Scale, Chinese version, where scores ranged from 0 to 100, with higher scores indicating a higher level of self-efficacy [40].

UI knowledge was assessed using the Chinese version of the Urinary Incontinence Quiz, which is widely used to measure UI-related knowledge. The Urinary Incontinence Quiz is a 15-item validated instrument, with a total score ranging from 0 to 15, with a higher score indicating better UI knowledge [41].

### Protocol Changes

We have made several modifications to the original protocol before trial onset. First, the inclusion criteria were changed to gain additional insight into the preventive effect of the UIW app–based intervention. Therefore, we included all pregnant women regardless of UI status and type of UI, instead of recruiting only pregnant women with stress UI (SUI), as planned at the study onset. In addition, the sample size estimation was changed accordingly. Second, the secondary outcomes were updated. We excluded the assessment of pelvic floor muscle strength (originally a secondary outcome) at 6 weeks post partum because of the poor acceptability of pelvic floor muscle surface electromyography among Chinese women at the time of recruitment, a measurement that requires the insertion of a vaginal probe into the vagina. We also replaced the risk factors for UI (originally a secondary outcome) with UI-related knowledge (supplementally a secondary outcome). In addition, after trial commencement, although we did our best to follow up as many participants as possible at 3 months and 6 months post partum (originally follow-up end points), the loss rate at 3 months post partum was higher than expected; therefore, outcomes were reported only at 2 months after randomization and 6 weeks post partum. The abovementioned changes were implemented before the data were processed, with no effect on trial implementation.

### Statistical Analysis

Sample size estimation was performed using G*Power (version 3.1; Heinrich-Heine-Universität Düsseldorf) [42]. As no previous study has assessed the effect of app-based UI management intervention in pregnant women regardless of UI status and type, a conservative medium effect size (0.25) was expected in this study [43]. With 80% power at the 5% significance level (2-sided), 43 participants were required in each group. Considering a potential dropout rate of 20%, the target sample size required at least 54 participants in each group (108 participants in total).

An intention-to-treat approach was used in all data analyses that included all participants. Baseline data are shown as means and SDs for continuous variables and as counts and percentages for categorical variables, with differences in randomization groups assessed using independent sample 2-tailed *t* tests and chi-square tests, respectively. Missing values during follow-ups were imputed using multiple imputations [44]. Variables used for imputation included age, education, prepregnancy BMI, number of pregnancies, abortion history, vaginal delivery history, cesarean section history, constipation, gestational week at birth, delivery mode, perineal injury, new birth weight, UI during pregnancy, and outcome values.

For the primary and secondary outcomes, an analysis of a linear mixed model was used to compare the effects of the intervention between groups, accounting for both repeated measures and individual heterogeneity (random effect) [45]. The models incorporated group, time, and interactions between group and time as fixed covariates and the participants as random intercepts, with adjustment for prepregnancy BMI, abortion history, delivery mode, and UI during pregnancy, based on a previous study [46].

An additional post hoc subgroup analysis was performed using tests for intervention-subgroup interactions to explore whether the primary outcome results differed in subsets of participants grouped by baseline characteristics.

Statistical analyses were performed using R software (version 4.2.1; R Foundation for Statistical Computing). A 2-sided *P* value of <.05 was considered statistically significant.

## Results

### Participant Characteristics

**Figure 1:**
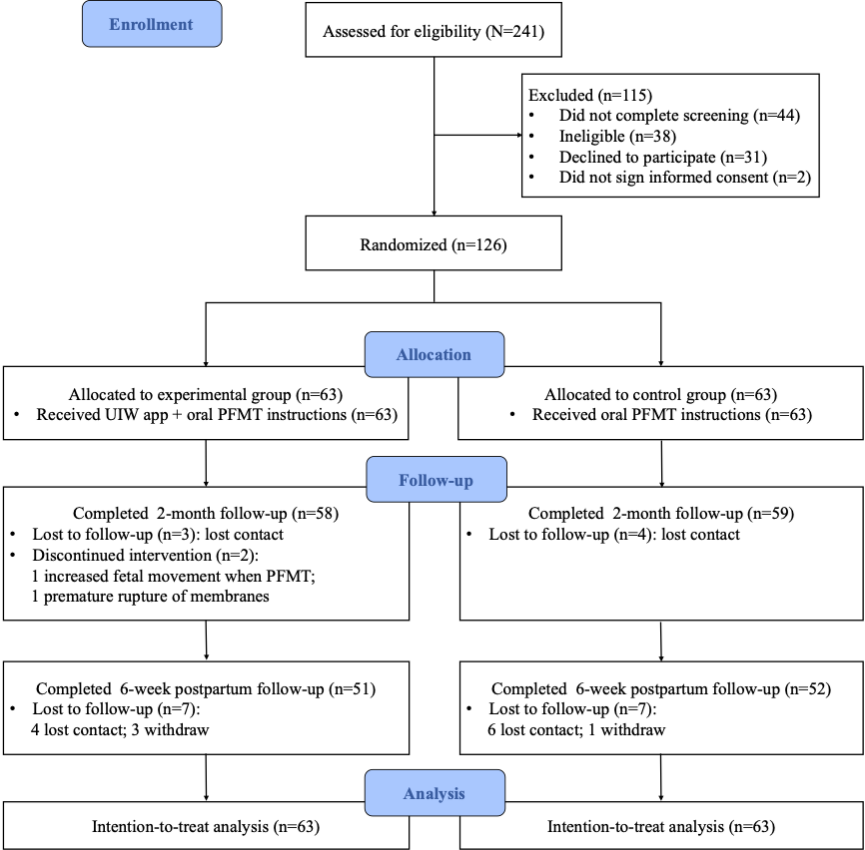
A CONSORT (Consolidated Standards of Reporting Trials) flowchart. PFMT: pelvic floor muscle training; UIW: Urinary Incontinence for Women.

The follow-up assessment ended in July 2021. The CONSORT (Consolidated Standards of Reporting Trials) diagram of the participant flow is shown in Figure 1. Of the 241 screened women, 126 (52.3%) met the inclusion criteria, provided informed consent, and completed the baseline assessment, and they were randomly assigned (1:1) to the experimental group (n=63) or the control group (n=63). Of the 126 participants, 117 (92.9%) women completed follow-up visits at 2 months after randomization, and 103 (81.7%) women completed follow-up visits at 6 weeks after delivery. The mean age of the 126 participants was 28.75 (SD 3.33) years; 68 (54%) participants had a bachelor’s degree or above, 110 (87.3%) participants delivered vaginally, and 87 (69%) participants reported experiencing urine leakage during pregnancy. The demographic, obstetrical characteristics, and outcomes of the participants at baseline were similar between the 2 groups (Table 1). No statistically significant differences were found in the baseline data between the participants who completed all follow-ups and those who did not (all *P*>.05), except that those lost to follow-up were more likely to have lower education levels (*P*=.01) and higher gestational age of delivery (*P*=.002; Multimedia Appendix 2).

**Table 1:** Baseline characteristics of participants in the experimental and control groups.

Characteristic		Total (n=126)	Experimental group (n=63)	Control group (n=63)	*P* value	
Age (years), mean (SD)		28.75 (3.33)	28.35 (3.60)	29.16 (3.01)	.17	
**Education level, n (%)**						
	Junior college and below	58 (46.0)	27 (43)	31 (49)	.47	
	Bachelor’s degree and above	68 (54.0)	36 (57)	32 (51)		
Prepregnancy BMI (kg/m^2^), mean (SD)		20.82 (2.68)	20.53 (2.53)	21.11 (2.81)	.23	
**Number of pregnancies, n (%)**						.23
	1	58 (46.0)	32 (51)	26 (41)		
	2	47 (37.3)	24 (38)	23 (37)		
	≥3	21 (16.7)	7 (11)	14 (22)		
Abortion history (yes), n (%)		24 (19.0)	10 (16)	14 (22)	.36	
Vaginal delivery history (yes), n (%)		56 (44.4)	25 (40)	31 (49)	.28	
Cesarean section history (yes), n (%)		8 (6.3)	3 (5)	5 (8)	.72	
Constipation (yes), n (%)		44 (34.9)	20 (32)	24 (38)	.46	
Gestational week at birth, mean (SD)		39.25 (1.09)	39.21 (1.05)	39.29 (1.13)	.68	
**Delivery mode, n (%)**						
	Vaginal delivery	110 (87.3)	56 (89)	54 (86)	.59	
	Cesarean section	16 (12.7)	7 (11)	9 (14)		
Perineal injury (yes), n (%)		101 (80.2)	49 (78)	52 (83)	.50	
New birth weight (g), mean (SD)		3231.75 (365.55)	3263.81 (338.19)	3199.68 (391.09)	.33	
UI^a^ during pregnancy (yes)^b^, n (%)		87 (69.0)	46 (73)	41 (65)	.34	
UI symptom severity (ICIQ-UI-SF^c^ score^d^), mean (SD)		4.64 (4.06)	4.98 (4.04)	4.30 (4.09)	.35	
Quality of life (Incontinence Impact Questionnaire-7 score^d^), mean (SD)		1.54 (2.41)	1.83 (2.02)	1.25 (2.72)	.18	
Self-efficacy with pelvic floor muscle training (Broome Pelvic Muscle Self-Efficacy Scale score^e^), mean (SD)		50.13 (21.43)	52.27 (16.94)	48.00 (25.09)	.27	
Knowledge of UI (Urinary Incontinence Quiz score^e^), mean (SD)		4.60 (3.35)	4.48 (3.23)	4.71 (3.48)	.69	

^a^UI: urinary incontinence.

^b^An International Consultation on Incontinence Questionnaire-Urinary Incontinence Short Form (ICIQ-UI-SF) score of 0 indicates no urinary incontinence during pregnancy, whereas a nonzero score indicates urinary incontinence during pregnancy.

^c^ICIQ-UI-SF: International Consultation on Incontinence Questionnaire-Urinary Incontinence Short Form.

^d^A higher score indicates a worse outcome.

^e^A higher score indicates a better outcome.

### Primary Outcome

**Figure 2:**
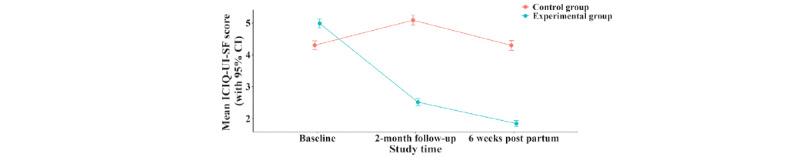
Urinary incontinence symptom severity changes over time for the experimental and control groups. ICIQ-UI-SF: International Consultation on Incontinence Questionnaire-Urinary Incontinence Short Form.

The results of the primary outcomes are shown in Figure 2 and Table 2. At 2 months after randomization, participants in the experimental group had significantly less severe UI symptoms (ICIQ-UI-SF score) compared with the control group (mean difference −2.86, 95% CI −4.09 to −1.64; *P*<.001). Similar results were observed at the 6 weeks postpartum follow-up, and between-group differences in the ICIQ-UI-SF score remained statistically significant (mean difference −2.68, 95% CI −3.87 to −1.49; *P*<.001). The linear mixed model also indicated that the interactions between the intervention and each follow-up time (at 2 months after randomization and 6 weeks post partum) were statistically significant (*P*<.001).

As shown in Figure 2, in the experimental group, the average score declined continuously from 4.98 at baseline to 1.85 at 6 weeks post partum, whereas in the control group, the average UI symptom severity score increased from 4.30 at baseline to 5.09 at 2 months after randomization and then declined to 4.30 at 6 weeks after delivery. These results did not substantially differ from the data gathered before replacing the missing data (Multimedia Appendix 2).

**Table 2:** Effects of the intervention on the primary outcome (urinary incontinence symptom severity) at 2 months after randomization and 6 weeks post partum.

Study time	Follow-up time, mean (SD)^a^		Between-group differences, mean (95% CI)	*P* value	Linear mixed-effect model results (*P* value^b^)		
	Experimental group (n=63)	Control group (n=63)			Time	Group	Group×time
Baseline	4.98 (4.01)	4.30 (4.05)	N/A^c^	N/A	N/A	.55	N/A
2 months after randomization	2.52 (3.26)	5.09 (4.35)	−2.86 (−4.09 to −1.64)	<.001	.02	N/A	<.001
6 weeks post partum	1.85 (2.66)	4.30 (4.38)	−2.68 (−3.87 to −1.49)	<.001	.99	N/A	<.001

^a^A higher score indicates a more severe urinary incontinence symptom.

^b^Adjusted for prepregnancy BMI, abortion history, delivery mode, and urinary incontinence during pregnancy.

^c^N/A: not applicable.

### Secondary Outcomes

The secondary outcomes are summarized in Table 3. At 2 months after randomization and 6 weeks post partum, participants in the experimental group had significantly increased quality of life (2 months after randomization: mean difference −0.85, 95% CI −1.52 to −0.18, *P*=.01; 6 weeks post partum: mean difference −2.19, 95% CI −3.15 to −1.23, *P*<.001), improved self-efficacy (2 months after randomization: mean difference 19.78, 95% CI 12.94 to 26.63, *P*<.001; 6 weeks post partum: mean difference 24.67, 95% CI 17.63 to 31.71, *P*<.001), and knowledge of UI (2 months after randomization: mean difference 3.54, 95% CI 2.58 to 4.51, *P*<.001; 6 weeks post partum: mean difference 3.64, 95% CI 2.62 to 4.66, *P*<.001) compared with the control group. For all secondary outcomes, the interactions between the intervention and time remained statistically significant (all *P*<.05; Multimedia Appendix 2 provides more details).

**Table 3:** Effects of the intervention on secondary outcomes (quality of life, self-efficacy with pelvic floor muscle training, and knowledge of urinary incontinence [UI]) at 2 months after randomization and 6 weeks post partum.

Outcomes measures		Within-group changes,^a,b^ mean (95% CI)		Between-group differences,^a^ mean (95% CI)	*P* value^a^
		Experimental group (n=63)	Control group (n=63)		
**Quality of life (Incontinence Impact Questionnaire-7 score^c^)**					
	2 months after randomization	−0.98 (−1.51 to −0.44)	0.31 (−0.63 to 1.26)	−0.85 (−1.52 to −0.18)	.01
	6 weeks post partum	−0.88 (−1.44 to −0.33)	1.74 (0.74 to 2.75)	−2.19 (−3.15 to −1.23)	<.001
**Self-efficacy with pelvic floor muscle training (Broome Pelvic Muscle Self-Efficacy Scale score^d^)**					
	2 months after randomization	16.94 (12.13 to 21.75)	1.49 (−6.62 to 9.59)	19.78 (12.94 to 26.63)	<.001
	6 weeks post partum	15.24 (10.30 to 20.18)	−4.99 (−13.31 to 3.32)	24.67 (17.63 to 31.71)	<.001
**Knowledge of UI (Urinary Incontinence Quiz score^d^)**					
	2 months after randomization	3.76 (2.82 to 4.70)	−0.18 (−1.25 to 0.89)	3.54 (2.58 to 4.51)	<.001
	6 weeks post partum	3.54 (2.56 to 4.52)	−0.38 (−1.47 to 0.71)	3.64 (2.62 to 4.66)	<.001

^a^Adjusted for prepregnancy BMI, abortion history, delivery mode, and urinary incontinence during pregnancy.

^b^Mean change between baseline and follow-up.

^c^A higher score indicates a worse outcome.

^d^A higher score indicates a better outcome.

### Subgroups Analyses

**Figure 3:**
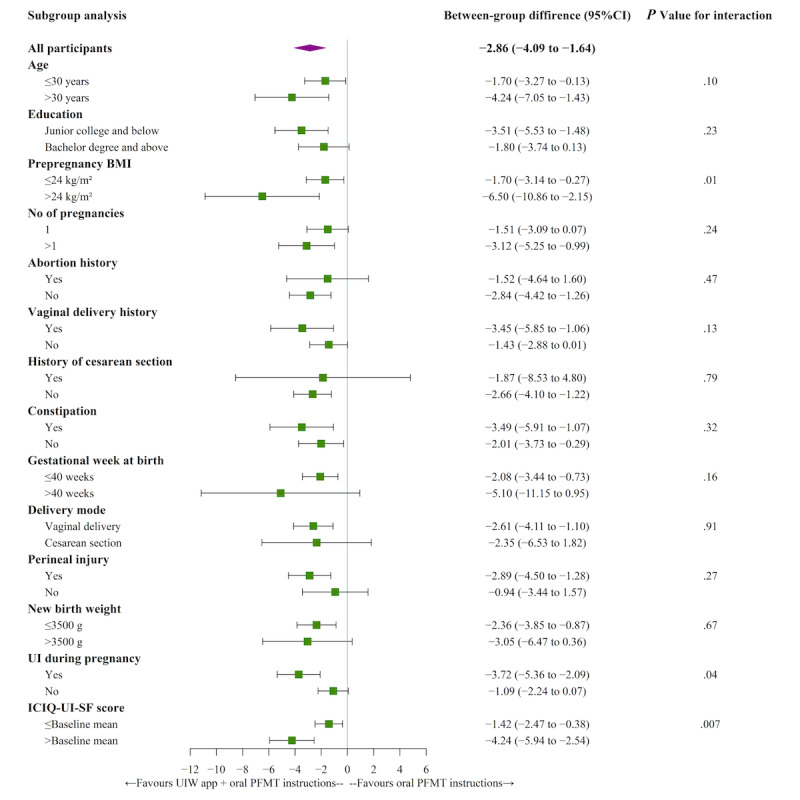
Subgroup analyses of primary outcome at 2 months after randomization. ICIQ-UI-SF: International Consultation on Incontinence Questionnaire-Urinary Incontinence Short Form; PFMT: pelvic floor muscle training; UI: urinary incontinence; UIW: Urinary Incontinence for Women.

**Figure 4:**
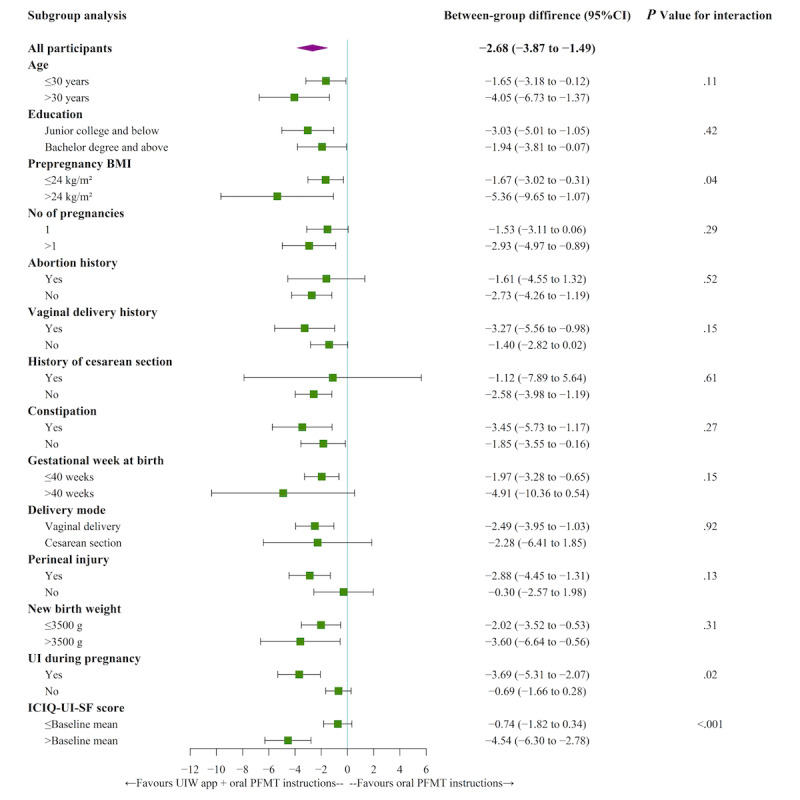
Subgroup analyses of primary outcome at 6 weeks post partum. ICIQ-UI-SF: International Consultation on Incontinence Questionnaire-Urinary Incontinence Short Form; PFMT: pelvic floor muscle training; UI: urinary incontinence; UIW: Urinary Incontinence for Women.

The post hoc subgroup analyses of the primary outcome revealed statistically significant interactions between subgroups of prepregnancy BMI, UI status during pregnancy, and baseline ICIQ-UI-SF scores, both at 2 months after randomization and 6 weeks post partum (Figures 3-4). Those who were overweight before pregnancy (prepregnancy BMI >24 kg/m^2^) had statistically significant improvement in UI symptom severity than those with normal prepregnancy BMI (2 months after randomization: *P*=.01 and 6 weeks post partum: *P*=.04). Furthermore, among participants with UI during pregnancy, the between-group difference in the intervention effect was significantly lower than among participants with non-UI during pregnancy (2 months after randomization: *P*=.04 and 6 weeks post partum: *P*=.02). Moreover, those with a higher baseline ICIQ-UI-SF score had statistically significant improvement in UI symptom severity than those with a lower baseline ICIQ-UI-SF score (2 months after randomization: *P*=.007 and 6 weeks post partum: *P*<.001).

### Adverse Events

There were no reported adverse events over the duration of the study.

## Discussion

### Principal Findings

To the best of our knowledge, this is the first trial that combines mHealth and PFMT to prevent and treat UI symptoms in Chinese pregnant women. The results of this study support the effectiveness of UIW app–based interventions in reducing UI symptom severity, UI impact on life, and enhancement of PFMT self-efficacy and UI-related knowledge among pregnant women. This could help to update the Cochrane review to determine the effect of antenatal PFMT as a mixed preventive and therapeutic strategy for UI.

Greater improvements in the severity of UI symptoms were observed at 2 months after randomization in the experimental group than in the control group, and the improvement was sustained at 6 weeks post partum. These findings were in line with those of previous studies conducted on community-dwelling women with SUI using app-based treatment programs compared with no treatment [47]. However, in contrast to our results, an intervention study conducted among Chinese primiparas comparing an app-based audio guidance PFMT intervention with conventional home-based training, where the latter was similar to the oral PFMT instructions from our trial, showed no significant difference [33]. Differences in the features of UI mobile apps could partly explain this discrepancy. For example, the app applied in the former study was a single audio guidance for PFMT, whereas in our study, it included audio as well as animation guidance, and multidimensional and figurative forms of information may be more likely to enhance participants’ understanding of PFMT, thus enhancing intervention outcomes. In addition, participants were followed up for 6 months post partum in the former study versus 6 weeks post partum in this study, which possibly attenuated the effect of the intervention.

The mean between-group differences in symptom severity score reduction (2.86 at 2 months after randomization; 2.68 at 6 weeks post partum) were slightly smaller than those achieved in similar studies using a mobile app for self-management of UI [47,48]. Therefore, we surmised that the observed differences might be related to the study populations recruited in our study (nonincontinent included), possibly reducing the treatment benefit because of the lower baseline UI symptom severity score. However, it is necessary to ensure that app-based UI self-management interventions are available to all pregnant women in need, as pelvic exercise advice is not routinely practiced [49]. Surprisingly, these differences were statistically significant and exceeded the reported minimal clinically important difference (1.58) specifically described for women with SUI after PFMT via an mHealth method [50], thus indicating that the UIW intervention might be a potentially effective digital support for UI self-management.

The sustained improvement in quality during daily life and self-efficacy with PFMT and UI-related knowledge in the experimental group also showed encouraging results. The beneficial effects may be because the UIW app is an evidence-based and multifunctional tool based on behavior change techniques [51]. This method is recommended by the National Institute for Health and Care Excellence for its effectiveness in facilitating behavior change [52] and is widely applied in mHealth interventions [53,54], which may lead to a more positive response to PFMT. For example, using the skills of goal and planning (eg, supporting individuals to formulate specific, achievable, PFMT-related goals) and shaping knowledge (eg, providing advice on the method of performing skills training) may promote confidence in women. In addition, the technique of natural consequence possibly increased awareness and UI-related knowledge by providing consequence-related information of performing PFMT.

Previous qualitative interviews also showed that the app could help increase individuals’ confidence in performing PFMT independently and their awareness of UI symptoms [27,29]. As is well-known, adherence to PFMT is a crucial prerequisite of its effectiveness [55], and existing evidence suggests that it can be predicted by self-efficacy [56,57] and affected by knowledge and awareness of the UI symptom [58]. PFMT under the supervision of medical staff may help improve adherence; however, it is expensive and impractical in China’s context of insufficient medical resources. Considering these clinical conditions, the positive self-efficacy and knowledge in this study were fundamentally important, indicating that the app may address the problem of the unmet clinical need for PFMT guidance and UI-related education among pregnant women and further improve adherence to PFMT.

### Limitations

This study has some limitations. First, there were no objective outcome measures. Although the primary outcome evaluation depended on self-reporting, which might have overestimated the improvement of incontinence, it may best represent incontinence from the patient’s perspective, and the self-reported instrument (ICIQ-UI-SF) has been validated previously and widely used in randomized controlled trials [59,60]. Second, the unblinded study design was unavoidable because of the nature of the mHealth intervention but also a limitation that could leave a potential for bias. Third, this study lacks long-term follow-up to determine the effects of the app-based PFMT program over time, because of the higher than expected lost-to-follow-up rate. Fourth, the results of the post hoc subgroup analyses in this study should be interpreted with caution because of their exploratory nature and unbalanced sample sizes.

### Implications for Future Practice

Given the high incidence of UI during pregnancy and post partum and its severe impact on quality of life, it is critical in clinical practice to provide effective PFMT guidance for pregnant women, which is a crucial component of the primary prevention of UI. This study showed that oral PFMT instructions in conjunction with an app-based PFMT program were more efficient than oral PFMT instructions alone in reducing UI symptom severity and enhancing quality of life in the short term, indicating that the UIW app may be an effective adjunctive tool and could be routinely offered to all pregnant women. However, this was a single-center study, limiting our findings’ generalizability. Therefore, future multicenter studies should be conducted to replicate and extend our findings. In addition, future studies should track study participants for a longer period to provide evidence on whether the effects of the app-based PFMT intervention were sustained.

### Conclusions

These results demonstrated that the UIW app–based mHealth intervention effectively reduced the severity of UI symptoms during late pregnancy, and the effect was sustained at 6 weeks post partum. In addition, participants receiving the app-based PFMT intervention also revealed higher levels of quality of life, self-efficacy with PFMT, and UI knowledge at follow-up periods. This trial suggested that mHealth interventions might be a promising approach for delivering UI management services among pregnant women.

## Appendix

Multimedia Appendix 1 CONSORT-eHEALTH checklist (version 1.6.1).

Multimedia Appendix 2 Supplementary tables and figures.

## Abbreviations

CONSORT: Consolidated Standards of Reporting Trials
CONSORT-eHEALTH: Consolidated Standards of Reporting Trials of Electronic and Mobile Health Applications and Online Telehealth
ICIQ-UI-SF: International Consultation on Incontinence Questionnaire-Urinary Incontinence Short Form
IIQ-7: Incontinence Impact Questionnaire-7
mHealth: mobile health
PFMT: pelvic floor muscle training
SUI: stress urinary incontinence
UI: urinary incontinence
UIW: Urinary Incontinence for Women

## Data Availability

The data sets used and analyzed during this study are available from the corresponding author upon reasonable request.
