# Supplementary material for: Effect of a Mobile App–Based Urinary Incontinence Self-Management Intervention Among Pregnant Women in China: Pragmatic Randomized Controlled Trial
Source: J Med Internet Res. 2023 Jun 27;25:e43528. doi: 10.2196/43528 (PMC10337423; doi:10.2196/43528)

**Supplementary Tables and Figures:**

**Table S1.** Baseline data of participants who completed all follow-ups versus who did not.

**Table S2.** Linear mixed model results of the primary and secondary outcomes.

**Table S3.** Linear mixed model results of the primary outcome before replacing missing data.

**Table S4.** Comparisons between groups in primary and secondary outcomes over the study periods.

**Table S5.** Comparisons between groups in primary outcome over the study periods before replacing missing data.

**Figure S1.** Secondary outcomes changed over time for the experimental and control groups. IIQ-7: Incontinence Impact Questionnaire-7; BPMSES: Broome Pelvic Muscle Self-Efficacy Scale; UIQ: Urinary Incontinence Quiz.

**Table S1.** Baseline data of participants who completed all follow-ups versus who did not.

| **Characteristic** | **Total**  **(N=126)** | **Completed follow-ups (n=103)** | **Lost to follow-up**  **(n=23)** | ***P* value** |
| --- | --- | --- | --- | --- |
| Age (years), mean (SD) | 28.75 (3.33) | 28.80 (3.23) | 28.57 (3.80) | .79 |
| **Education level, n (%)** |  |  |  |  |
| Junior college and below | 58 (46.0) | 42 (40.8) | 16 (69.6) | .01 |
| Bachelor’s degree and above | 68 (54.0) | 61 (59.2) | 7 (30.4) |  |
| Prepregnancy BMI (kg/m^2^), mean (SD) | 20.82 (2.68) | 20.83 (2.71) | 20.77 (2.57) | .92 |
| **Number of pregnancies, n (%)** |  |  |  |  |
| 1 | 58 (46.0) | 46 (44.7) | 12 (52.2) | .78 |
| 2 | 47 (37.3) | 39 (37.9) | 8 (34.8) |  |
| ≥3 | 21 (16.7) | 18 (17.5) | 3 (13.0) |  |
| Abortion history (yes), n (%) | 24 (19.0) | 21 (20.4) | 3 (13.0) | .61 |
| Vaginal delivery history (yes), n (%) | 56 (44.4) | 46 (44.7) | 10 (43.5) | .92 |
| Cesarean section history (yes), n (%) | 8 (6.3) | 7 (6.8) | 1 (4.3) | .66 |
| Constipation (yes), n (%) | 44 (34.9) | 36 (35.0) | 8 (34.8) | .99 |
| Gestational week at birth, mean (SD) | 39.25 (1.09) | 39.14 (1.13) | 39.74 (0.69) | .002 |
| **Delivery mode, n (%)** |  |  |  |  |
| Vaginal delivery | 110 (87.3) | 92 (89.3) | 18 (78.3) | .27 |
| Cesarean section | 16 (12.7) | 11 (10.7) | 5 (21.7) |  |
| Perineal injury (yes), n (%) | 101 (80.2) | 82 (79.6) | 19 (82.6) | .97 |
| New birth weight (g), mean (SD) | 3231.75 (365.55) | 3209.03 (355.19) | 3333.48 (401.36) | .18 |
| UI during pregnancy (yes) ^a^, n (%) | 87 (69.0) | 70 (68.0) | 17 (73.9) | .58 |
| UI symptom severity (ICIQ-UI-SF score ^b^), mean (SD) | 4.64 (4.06) | 4.42 (3.82) | 5.65 (4.96) | .27 |
| Quality of life (IIQ-7 score ^b^), mean (SD) | 1.54 (2.41) | 1.45 (2.23) | 1.96 (3.10) | .46 |
| Self-efficacy with PFMT (BPMSES score ^c^), mean (SD) | 50.13 (21.43) | 52.00 (20.36) | 41.78 (24.44) | .07 |
| Knowledge of UI (UIQ score ^c^), mean (SD) | 4.60 (3.35) | 4.61 (3.42) | 4.52 (3.09) | .90 |
| **Abbreviations:** UI: urinary incontinence; PFMT: pelvic floor muscle training; ICIQ-UI-SF: International Consultation on Incontinence Questionnaire-Urinary Incontinence Short Form; IIQ-7: Incontinence Impact Questionnaire-7; BPMSES: Broome Pelvic Muscle Self-Efficacy Scale; UIQ: Urinary Incontinence Quiz.  ^a^ The ICIQ-UI-SF score of 0 indicates no UI during pregnancy, whereas a non-zero indicates UI during pregnancy.  ^b^ A higher score indicates a worse outcome.  ^c^ A higher score indicates a better outcome. | | | | |

**Table S2.** Linear mixed model results of the primary and secondary outcomes ^a^.

| **Variables** | **Beta coefficient** | **Standard error** | **95% CI** | ***P* value** |
| --- | --- | --- | --- | --- |
| UI symptom severity (ICIQ-UI-SF score ^b^) |  |  |  |  |
| Intercept | 3.84 | 2.13 | -0.34 to 8.03 | .07 |
| Group | 0.33 | 0.56 | -0.77 to 1.43 | .55 |
| 2 months after randomization vs BL | 0.78 | 0.34 | 0.12 to 1.44 | .02 |
| 6 weeks post partum vs BL | -0.004 | 0.38 | -0.75 to 0.74 | .99 |
| Group × (2 months after randomizationvs BL) | -3.25 | 0.48 | -4.19 to -2.32 | <.001 |
| Group × (6 weeks post partum vs BL) | -3.13 | 0.50 | -4.12 to -2.14 | <.001 |
| Quality of life (IIQ-7 score ^b^) |  |  |  |  |
| Intercept | 1.54 | 1.32 | -1.07 to 4.14 | .25 |
| Group | 0.40 | 0.39 | -0.37 to 1.18 | .31 |
| 2 months after randomization vs BL | 0.31 | 0.31 | -0.30 to 0.93 | .32 |
| 6 weeks post partum vs BL | 1.74 | 0.36 | 1.04 to 2.45 | <.001 |
| Group × (2 months after randomization vs BL) | -1.29 | 0.44 | -2.16 to -0.42 | .004 |
| Group × (6 weeks post partum vs BL) | -2.63 | 0.47 | -3.56 to -1.69 | <.001 |
| Self-efficacy with PFMT (BPMSES score ^c^) |  |  |  |  |
| Intercept | 54.87 | 13.83 | 27.66 to 82.07 | <.001 |
| Group | 4.66 | 3.49 | -2.21 to 11.53 | .18 |
| 2 months after randomization vs BL | 1.49 | 1.74 | -1.94 to 4.92 | .39 |
| 6 weeks post partum vs BL | -4.99 | 1.98 | -8.90 to -1.09 | .01 |
| Group × (2 months after randomization vs BL) | 15.45 | 2.49 | 10.55 to 20.35 | <.001 |
| Group × (6 weeks post partum vs BL) | 20.23 | 2.69 | 14.93 to 25.53 | <.001 |
| Knowledge of UI (UIQ score ^c^) |  |  |  |  |
| Intercept | 5.16 | 1.82 | 1.59 to 8.74 | .005 |
| Group | -0.35 | 0.52 | -1.37 to 0.67 | .50 |
| 2 months after randomization vs BL | -0.18 | 0.39 | -0.94 to 0.58 | .64 |
| 6 weeks post partum vs BL | -0.38 | 0.40 | -1.17 to 0.41 | .34 |
| Group × (2 months after randomization vs BL) | 3.94 | 0.54 | 2.87 to 5.01 | <.001 |
| Group × (6 weeks post partum vs BL) | 3.92 | 0.56 | 2.81 to 5.03 | <.001 |
| **Abbreviations:** UI: urinary incontinence; PFMT: pelvic floor muscle training; ICIQ-UI-SF: International Consultation on Incontinence Questionnaire-Urinary Incontinence Short Form; IIQ-7: Incontinence Impact Questionnaire-7; BPMSES: Broome Pelvic Muscle Self-Efficacy Scale; UIQ: Urinary Incontinence Quiz.  ^a^ Adjusted for prepregnancy BMI, abortion history, delivery mode, UI during pregnancy.  ^b^ A higher score indicates a worse outcome.  ^c^ A higher score indicates a better outcome.  **Explanation of the model:** Intercept, mean value in control group at baseline; Group, between-group differences at baseline; 2 months after randomization vs BL, difference from baseline in control group at 2 months after randomization; 6 weeks post partum vs BL, difference from baseline in control group at 6 weeks post partum; Group × (2 months after randomization or 6 weeks post partum vs BL), interaction effect, representing between-group differences from baseline. | | | | |

**Table S3.** Linear mixed model results of the primary outcome before replacing missing data ^a^.

| **Variables** | **Beta coefficient** | **Standard error** | **95% CI** | ***P* value** |
| --- | --- | --- | --- | --- |
| UI symptom severity (ICIQ-UI-SF score ^b^) |  |  |  |  |
| Intercept | 4.00 | 2.19 | -0.23 to 8.22 | .07 |
| Group | 0.30 | 0.57 | -0.79 to 1.39 | .59 |
| 2 months after randomization vs BL | 0.78 | 0.32 | 0.16 to 1.41 | .02 |
| 6 weeks post partum vs BL | 0.25 | 0.33 | -0.41 to 0.90 | .46 |
| Group × (2 months after randomization vs BL) | -3.32 | 0.45 | -4.20 to -2.43 | <.001 |
| Group × (6 weeks post partum vs BL) | -3.51 | 0.48 | -4.44 to -2.58 | <.001 |
| **Abbreviations:** UI: urinary incontinence; ICIQ-UI-SF: International Consultation on Incontinence Questionnaire-Urinary Incontinence Short Form.  ^a^ Adjusted for prepregnancy BMI, abortion history, delivery mode, UI during pregnancy.  ^b^ A higher score indicates a worse outcome.  **Explanation of the model:** Intercept, mean value in control group at baseline; Group, between-group differences at baseline; 2 months after randomization vs BL, difference from baseline in control group at 2 months after randomization; 6 weeks post partum vs BL, difference from baseline in control group at 6 weeks post partum; Group × (2 months after randomization or 6 weeks post partum vs BL), interaction effect, representing between-group differences from baseline. | | | | |

**Table S4.** Comparisons between groups in primary and secondary outcomes over the study periods ^a^.

| **Outcomes measures** | **Experimental group (n=63)** | |  | **Control group (n=63)** | | **Between-group differences,**  **mean (95% CI)** | ***P* value** |
| --- | --- | --- | --- | --- | --- | --- | --- |
|  | **Follow-up time, Mean (SD)** | **Within-group changes ^b^, mean (95% CI)** |  | **Follow-up time, Mean (SD)** | **Within-group changes ^b^, mean (95% CI)** |  |  |
| UI symptom severity (ICIQ-UI-SF score ^c^) |  |  |  |  |  |  |  |
| Baseline | 4.98 (4.01) |  |  | 4.30 (4.05) |  |  |  |
| 2 months after randomization | 2.52 (3.26) | -2.47 (-3.48 to -1.45) |  | 5.09 (4.35) | 0.78 (-0.36 to 1.93) | -2.86 (-4.09 to -1.64) | <.001 |
| 6 weeks post partum | 1.85 (2.66) | -3.14 (-4.16 to -2.12) |  | 4.30 (4.38) | -0.004 (-1.20 to 1.19) | -2.68 (-3.87 to -1.49) | <.001 |
| Quality of life (IIQ-7 score ^c^) |  |  |  |  |  |  |  |
| Baseline | 1.83 (2.00) |  |  | 1.25 (2.70) |  |  |  |
| 2 months after randomization | 0.85 (1.37) | -0.98 (-1.51 to -0.44) |  | 1.57 (2.38) | 0.31 (-0.63 to 1.26) | -0.85 (-1.52 to -0.18) | .01 |
| 6 weeks post partum | 0.94 (1.44) | -0.88 (-1.44 to -0.33) |  | 3.00 (3.56) | 1.74 (0.74 to 2.75) | -2.19 (-3.15 to -1.23) | <.001 |
| Self-efficacy with PFMT (BPMSES score ^d^) |  |  |  |  |  |  |  |
| Baseline | 52.27 (16.8) |  |  | 48.00 (24.89) |  |  |  |
| 2 months after randomization | 69.21 (11.99) | 16.94 (12.13 to 21.75) |  | 49.49 (23.83) | 1.49 (-6.62 to 9.59) | 19.78 (12.94 to 26.63) | <.001 |
| 6 weeks post partum | 67.51 (10.32) | 15.24 (10.30 to 20.18) |  | 43.01 (24.64) | -4.99 (-13.31 to 3.32) | 24.67 (17.63 to 31.71) | <.001 |
| Knowledge of UI (UIQ score ^d^) |  |  |  |  |  |  |  |
| Baseline | 4.48 (3.21) |  |  | 4.71 (3.45) |  |  |  |
| 2 months after randomization | 8.24 (2.35) | 3.76 (2.82 to 4.70) |  | 4.54 (2.90) | -0.18 (-1.25 to 0.89) | 3.54 (2.58 to 4.51) | <.001 |
| 6 weeks post partum | 8.01 (2.36) | 3.54 (2.56 to 4.52) |  | 4.33 (2.77) | -0.38 (-1.47 to 0.71) | 3.64 (2.62 to 4.66) | <.001 |
| **Abbreviations:** UI: urinary incontinence; PFMT: pelvic floor muscle training; ICIQ-UI-SF: International Consultation on Incontinence Questionnaire-Urinary Incontinence Short Form; IIQ-7: Incontinence Impact Questionnaire-7; BPMSES: Broome Pelvic Muscle Self-Efficacy Scale; UIQ: Urinary Incontinence Quiz.  ^a^ Adjusted for prepregnancy BMI, abortion history, delivery mode, UI during pregnancy.  ^b^ Indicates mean change between baseline and follow-up.  ^c^ A higher score indicates a worse outcome.  ^d^ A higher score indicates a better outcome. | | | | | | | |

**Table S5.** Comparisons between groups in primary outcome over the study periods before replacing missing data ^a^.

| **Outcomes measures** |  | **Experimental group (n=63)** | |  |  | **Control group (n=63)** | | **Between-group differences,**  **mean (95% CI)** | ***P* value** |
| --- | --- | --- | --- | --- | --- | --- | --- | --- | --- |
|  | **N** | **Follow-up time, Mean (SD)** | **Within-group changes ^b^, mean (95% CI)** |  | **N** | **Follow-up time, Mean (SD)** | **Within-group changes ^b^, mean (95% CI)** |  |  |
| UI symptom severity (ICIQ-UI-SF score ^c^) |  |  |  |  |  |  |  |  |  |
| Baseline | 63 | 4.98 (4.04) |  |  | 63 | 4.30 (4.09) |  | 0.15 (-0.80 to 1.10) | .76 |
| 2 months after randomization | 58 | 2.47 (3.23) | -2.59 (-3.61 to -1.58) |  | 59 | 4.98 (4.48) | 0.83 (-0.32 to 1.99) | -2.96 (-4.23 to -1.69) | <.001 |
| 6 weeks post partum | 51 | 2.00 (2.70) | -3.15 (-4.21 to -2.10) |  | 52 | 3.83 (4.44) | -0.14 (-1.34 to 1.06) | -2.40 (-3.65 to -1.14) | <.001 |
| **Abbreviations:** UI: urinary incontinence; ICIQ-UI-SF: International Consultation on Incontinence Questionnaire-Urinary Incontinence Short Form.  ^a^ Adjusted for prepregnancy BMI, abortion history, delivery mode, UI during pregnancy.  ^b^ Indicates mean change between baseline and follow-up.  ^c^ A higher score indicates a worse outcome. | | | | | | | | | |

**Figure S1.** Secondary outcomes changed over time for the experimental and control groups. IIQ-7: Incontinence Impact Questionnaire-7; BPMSES: Broome Pelvic Muscle Self-Efficacy Scale; UIQ: Urinary Incontinence Quiz.


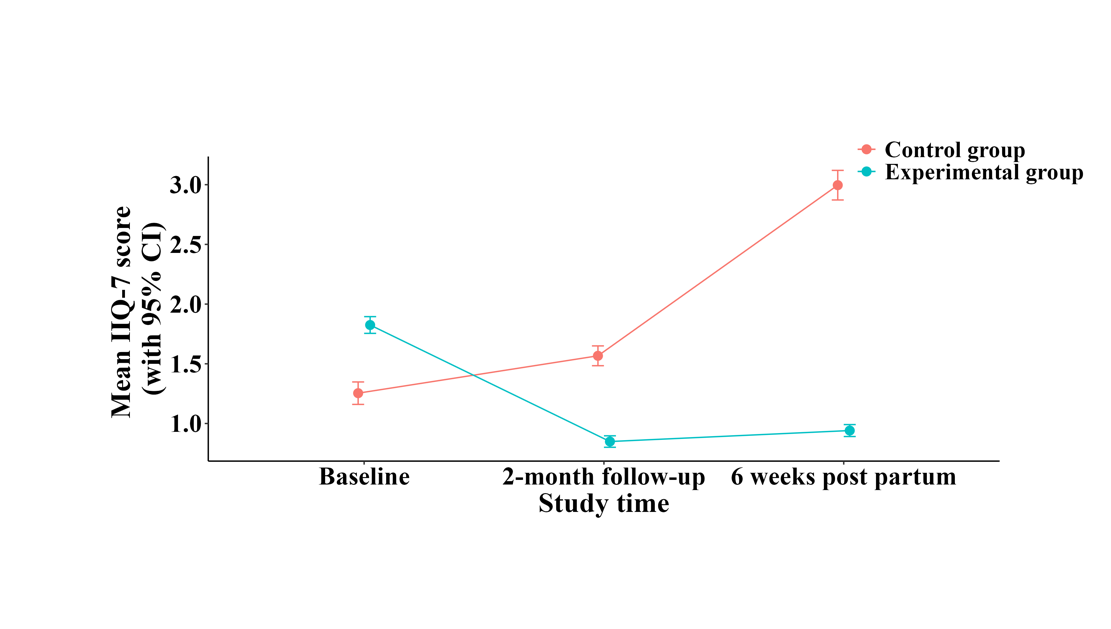

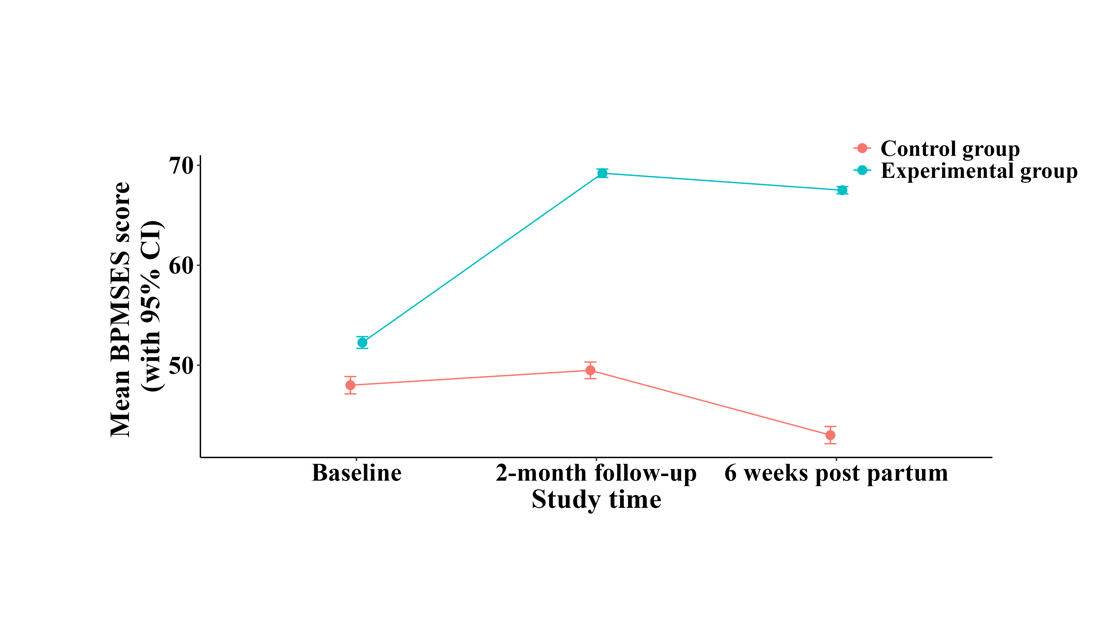

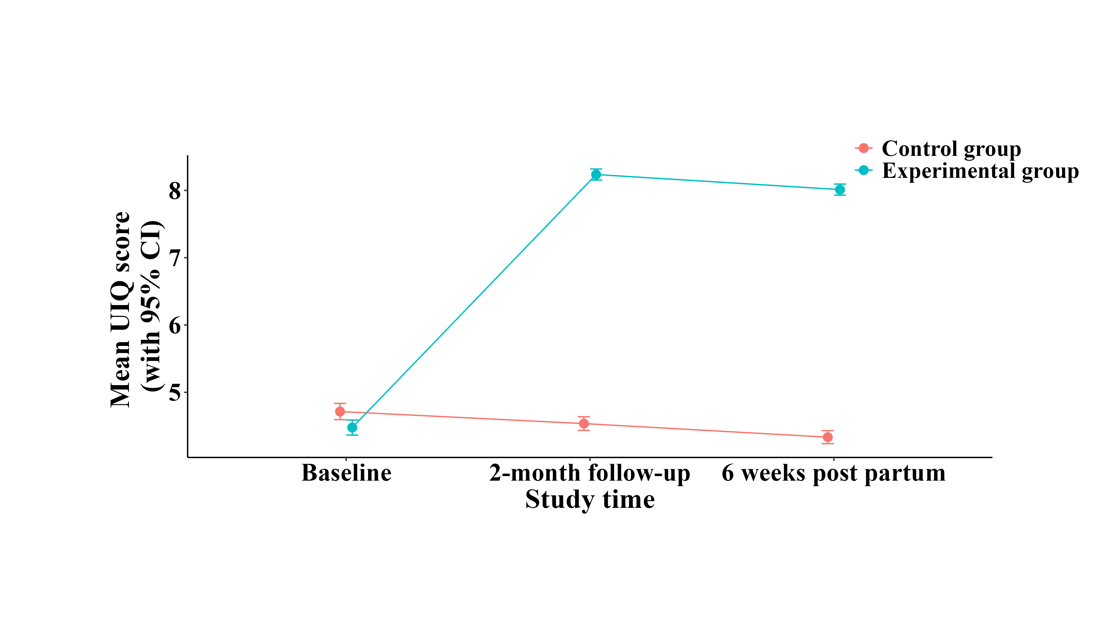

Supplement: Multimedia Appendix 2 [file jmir_v25i1e43528_app2.docx]
